# Crosstalk Between SUMO and Ubiquitin-Like Proteins: Implication for Antiviral Defense

**DOI:** 10.3389/fcell.2021.671067

**Published:** 2021-04-21

**Authors:** Mounira K. Chelbi-Alix, Pierre Thibault

**Affiliations:** ^1^INSERM UMR-S 1124, Université Paris Descartes, Paris, France; ^2^Institute for Research in Immunology and Cancer, Montréal, QC, Canada; ^3^Department of Chemistry, University of Montreal, Montréal, QC, Canada

**Keywords:** ubiquitin, SUMO, antiviral defense, interferon, restriction factors, ISG15, PML, TRIM25

## Abstract

Interferon (IFN) is a crucial first line of defense against viral infection. This cytokine induces the expression of several IFN-Stimulated Genes (ISGs), some of which act as restriction factors. Upon IFN stimulation, cells also express ISG15 and SUMO, two key ubiquitin-like (Ubl) modifiers that play important roles in the antiviral response. IFN itself increases the global cellular SUMOylation in a PML-dependent manner. Mass spectrometry-based proteomics enables the large-scale identification of Ubl protein conjugates to determine the sites of modification and the quantitative changes in protein abundance. Importantly, a key difference amongst SUMO paralogs is the ability of SUMO2/3 to form poly-SUMO chains that recruit SUMO ubiquitin ligases such RING finger protein RNF4 and RNF111, thus resulting in the proteasomal degradation of conjugated substrates. Crosstalk between poly-SUMOylation and ISG15 has been reported recently, where increased poly-SUMOylation in response to IFN enhances IFN-induced ISGylation, stabilizes several ISG products in a TRIM25-dependent fashion, and results in enhanced IFN-induced antiviral activities. This contribution will highlight the relevance of the global SUMO proteome and the crosstalk between SUMO, ubiquitin and ISG15 in controlling both the stability and function of specific restriction factors that mediate IFN antiviral defense.

## Introduction

The establishment and maintenance of the innate antiviral response in cells is defined by the function of interferons (IFNs), important cytokines that were originally discovered by Isaacs and Lindenmann (1957). While they are mostly known for their antiviral activity, they also have important immunomodulatory, anti-proliferative and apoptotic activities (Borden et al., 2007; Chelbi-Alix and Wietzerbin, 2007). Based on the type of receptor through which they signal, human IFNs are classified into multiple type I species (including IFNα and IFNβ), one type II (IFNγ) and four members of type III species (IFNλ1-4) (Chelbi-Alix and Wietzerbin, 2007).

The innate immune system constitutes the first line of host defense during viral infection triggering IFN synthesis and establishing an antiviral state. IFNs are secreted and bind to their cognate receptors (IFNAR for IFNα/β, IFNGR for IFNγ, or IFNLR for IFNλ), and subsequently activate the JAK/STAT pathways to trigger the transcription of more than one hundred IFN-Stimulated Genes (ISGs) (Schneider et al., 2014). Some of these ISG products act as restriction factors blocking various steps of the viral life cycle while others are positive or negative regulators of the IFN pathway. The complex regulation of IFN activities is not only essential to ensure a strong antiviral response but is also required to elicit a return to cell homeostasis.

Briefly, type I and type III IFNs bind to their respective receptors and activate Janus kinase 1 (JAK1) and Tyrosine kinase 2 (TYK2), which phosphorylate Signal Transducer and Activator of Transcription proteins (STAT1 and STAT2) (Barrat et al., 2019). Phosphorylated STATs heterodimerize (pSTAT1:pSTAT2) or homodimerize (pSTAT2:pSTAT2) and form with the DNA binding protein IFN Regulatory Factor (IRF9), the IFN-Stimulated Gene Factor 3 (ISGF3) complex. ISGF3 translocates into the nucleus to induce ISGs containing in their promoters an IFN-Stimulated Response Element (ISRE). The interaction of type II IFN to its specific receptor induces the phosphorylation of STAT1 by JAK1 and JAK2, the homodimerization of pSTAT1, their migration to the nucleus to induce ISGs containing in their promoters a Gamma-Activated Sequence (GAS).

Interestingly, STAT1 SUMOylation inhibits IFN-induced STAT1 phosphorylation (pSTAT1) resulting in a decrease of IFNγ-induced transcription without affecting the activity of IFNα since pSTAT2 homodimers can compensate for the lack of pSTAT1 (Ungureanu et al., 2005; Maarifi et al., 2015). Accordingly, several reports indicate the existence of alternative STAT2 signaling pathways in response to type I IFN that are independent from STAT1 (Blaszczyk et al., 2015; Rengachari et al., 2018). Of note, either ISGylation or ubiquitylation can modify lysine residues of STAT1, suggesting a possible interplay between these ubiquitin-like (Ubl) proteins in the regulation of STAT1 activity. While STAT1 ISGylation maintains the levels of pSTAT1 and promotes STAT1 association with ProMyelocytic Leukemia (PML) nuclear bodies (NBs) (Fan et al., 2020), decreased levels of ISGylation lead to an increase in STAT1 poly-ubiquitylation, and its degradation by the proteasome (Ganesan et al., 2016).

Several ISG products act as restriction factors mediating the IFN-induced innate immune antiviral response. When the restriction factors are constitutively expressed, they mediate the intrinsic antiviral activity, which confer viral resistance in the absence of IFNs (Hannoun et al., 2016; Wang et al., 2017a). The restriction factors are also further induced upon viral infection or IFN treatment, and individually they can interfere with a particular stage of the viral life cycle (El Asmi et al., 2018). Interestingly, some restriction factors are conjugated to SUMO and their modifications are further enhanced in response to IFN and also required for their antiviral activity (Geoffroy and Chelbi-Alix, 2011; Marcos-Villar et al., 2013; Hannoun et al., 2016; El-Asmi et al., 2020a).

Interferon response is achieved in part through protein modifications of its key players. In particular, SUMOylation and ISGylation are important Ubls implicated in intrinsic and innate immunity regulating IFN production and IFN signaling as well as localization, stability and activity of many restriction factors (Maarifi et al., 2015; Perng and Lenschow, 2018; Dzimianski et al., 2019; El-Asmi et al., 2020b). Whereas the expression of ISG15 is directly induced by IFNs (Loeb and Haas, 1992), that of SUMO1/2/3 is enhanced by IFNs through a miRNA-based mechanism involving the Lin28/let-7 axis (Sahin et al., 2014).

SUMOylation emerged as a key regulator during IFN treatment or viral infection with a drastic change in the SUMO proteome (Domingues et al., 2015; Sloan et al., 2015; Maroui et al., 2018; El-Asmi et al., 2020a). The increase in IFN response of the level of cellular SUMOylation and the SUMOylation of several restriction factors contribute to the antiviral function of the host (Hannoun et al., 2016; El-Asmi et al., 2020a). In contrast, viruses have developed various strategies to usurp SUMO host pathway targeting each step of SUMOylation process for their own benefit and to the detriment of the host. SUMO either directly targets viral proteins or alters the expression and function of cellular proteins implicated in antiviral defense. Exploitation of the SUMO pathway by DNA and RNA viruses has been reported in several reviews (Everett et al., 2013; Wimmer and Schreiner, 2015; Lowrey et al., 2017; El Motiam et al., 2020).

Importantly, IFN was shown to enhance levels of cellular SUMOylation (Maroui et al., 2018), ubiquitylation (Seifert et al., 2010) and ISGylation (Harty et al., 2009). In addition, increased poly-SUMOylation further enhances ubiquitylation and ISGylation in reponse to IFN resulting in either dowregulation or upregulation of several ISG products (El-Asmi et al., 2020b).

In this review, we will focus on the global SUMO proteome analysis in response to IFN and the consequence of the interplay between SUMOylation, ubiquitylation and ISGylation on IFN-induced antiviral defense.

## Proteome-Wide Analysis of Protein Sumoylation

**FIGURE 1:**
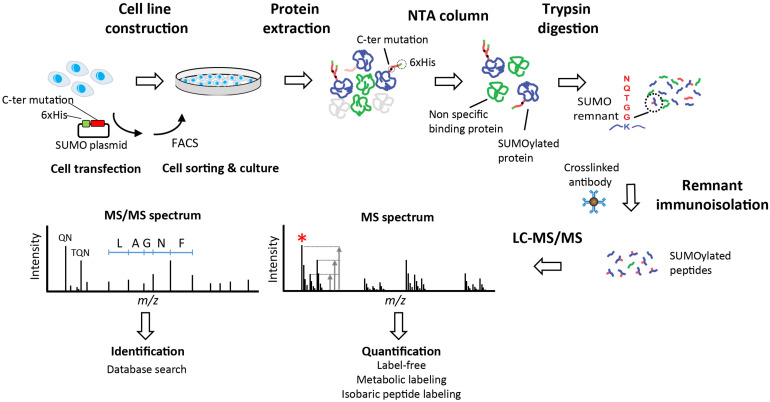
Identification of SUMOylated proteins. Stable cell lines are generated using plasmids that facilitate the immunoisolation of conjugated substrates. Plasmid incorporate a 6xHis tag for the enrichment of SUMOylated proteins using immobilized metal affinity chromatography with Ni-NTA resin. Cells are lysed and proteins are extracted prior to purification of SUMOylated proteins on Ni-NTA beads. Proteins are digested with trypsin directly on beads and tryptic peptides with remnant SUMO are isolated by immunoaffinity using a specific antibody. Purified SUMO peptides are analyzed by LC-MS/MS to obtain a survey scan (MS spectrum) from which peptide ions are selected for MS/MS sequencing (red asterisk). The ratio of peptide ion intensities from the MS spectrum is used to determine the relative changes in abundance between conditions (Quantification). Different quantification strategies including label-free, metabolic labeling (e.g., stable isotope labeling in cell culture, SILAC) or isobaric peptide labeling can be used to determine changes in protein abundance. Fragment ions observed in the MS/MS spectrum are used to identify the peptide sequence and the cognate protein with one of several database search engines (Identification). Specific fragment ions associated with the SUMO remnant side chains are observed in the MS/MS spectrum (QN at *m/z* 243 and TQN at *m/z* 344) are used to confirm the identification of SUMOylated peptides.

Different approaches including overexpression, knockdown, or knockout of either SUMO itself or the sole SUMO conjugating enzyme Ubc9 are used to understand the role of SUMOylation and to identify SUMO conjugates under different cell stimulation. However, the system-wide analysis of protein SUMOylation requires efficient affinity purification and sensitive mass spectrometry (MS) methods in view of the low abundance and rapid turnover of this modification. To identify and quantify protein SUMOylation in a global and site-specific manner, most methods rely on a two-step approach where SUMOylated proteins are first isolated by affinity purification, then digested with trypsin, and the modified peptides are subsequently purified by immunoaffinity precipitation (Figure 1) (Hendriks et al., 2014; Impens et al., 2014; Lamoliatte et al., 2014; Tammsalu et al., 2015). Exogenous expression of SUMO mutants containing a N-terminal poly-histidine tag is typically used to purify SUMO-modified proteins on Ni-NTA beads. In contrast to other Ubl such as ubiquitin, NEDD8 and ISG15, the tryptic digestion of SUMOylated proteins gives rise to large (26–32 amino acids) remnants that are difficult to sequence by MS. To facilitate peptide sequencing and SUMO peptide immunoprecipitation, an arginine residue is inserted near the C-terminus of SUMO to create smaller remnants upon tryptic digestion. Purified SUMOylated peptides can then by analyzed by MS to identify the protein substrates, the sites of modification and the type of SUMO chains appended to conjugates. Previous studies used SUMO remnant affinity purification in combination with quantitative proteomics to profile the changes in protein SUMOylation upon different cell stimuli including arsenic trioxide (As_2_O_3_) (Galisson et al., 2011; Rinfret Robert et al., 2020), proteasome inhibition (Hendriks et al., 2014; Lamoliatte et al., 2014), heat shock (Liebelt et al., 2019) and IFN (Maroui et al., 2018; El-Asmi et al., 2020a,b).

It is noteworthy that ubiquitylated substrates display a diglycine remnant left on the side-chain lysine after trypsin digestion, and antibodies recognizing the corresponding diglycine motif are used for immunoaffinity purification (Xu et al., 2010; Kim et al., 2011). Building upon these technical advances, a sequential Ubl remnant immunoaffinity approach was introduced to identify substrates modified by both ubiquitin and SUMO (Lamoliatte et al., 2017). This latter approach enabled the identification of more than 10,000 SUMO sites and revealed important crosstalk between ubiquitin and SUMO including the SUMOylation of proteasome subunits, a modification required for the recruitment of proteasome to PML NBs.

## PML, a Key Player in IFN Response and SUMO Pathway

ProMyelocytic leukemia protein, also known as TRIM19, is found in the nucleoplasm and assemble as PML NBs in the nuclear matrix. PML NBs are dynamic structures harboring a few permanent resident proteins (e.g., PML, Sp100, and SUMO) and numerous transient proteins depending on cell stimulation (i.e., stress, IFN, or viral infection) (van Damme et al., 2010; Geoffroy and Chelbi-Alix, 2011). PML NBs are implicated in various processus including stress response, apoptosis, protein degradation, viral infection and IFN response (Regad and Chelbi-Alix, 2001; Everett and Chelbi-Alix, 2007; Bernardi et al., 2008; Geoffroy and Chelbi-Alix, 2011; Nisole et al., 2013).

Several PML isoforms (PMLI to PMLVII) are generated from a single *PML* gene by alternative splicing. They share the same N-terminal region containing the RBCC/TRIM motif but differ in their C-terminus. The variability in their COOH-terminal region leads to the specific functions of some PML isoforms (Jensen et al., 2001; Maroui et al., 2011; Reichelt et al., 2011; Nisole et al., 2013).

ProMyelocytic leukemia protein emerges as a significant co-regulator of the IFN pathway. PML positively regulates virus-induced IFN synthesis (El Asmi et al., 2014) as well as type I and type II IFN signaling (El Bougrini et al., 2011; Kim and Ahn, 2015). Also, PML acts as a restriction factor in intrinsic and innate host antiviral defense both requiring its SUMOylation (Everett and Chelbi-Alix, 2007; Geoffroy and Chelbi-Alix, 2011; El Asmi et al., 2014). Key associations have been reported between PML NBs, IFN, antiviral defense and SUMO pathway.

### PML and IFN Response

All IFNs directly induce the *PML* gene *via* the ISRE and the GAS motifs present in its promoter resulting in the increase of PML isoforms (Chelbi-Alix et al., 1995; Stadler et al., 1995). Analysis of *PML* knockout mice and their derived cell lines (PML−/−) shows that they are more susceptible to viral infections (Blondel et al., 2002; Bonilla et al., 2002; El McHichi et al., 2010). Also, PML−/− cells are defective in IFN signaling and in the induction of apoptosis induced by type I and type II IFNs (Wang et al., 1998; El Bougrini et al., 2011).

It is well established that several infections with DNA and RNA viruses result in PML degradation and/or PML NB disorganization (Everett and Maul, 1994; Chelbi-Alix and de The, 1999; Muller and Dejean, 1999; Everett and Chelbi-Alix, 2007; Tavalai and Stamminger, 2008; El McHichi et al., 2010; Geoffroy and Chelbi-Alix, 2011), suggesting that PML NB alteration could be a viral strategy to evade cellular resistance mechanisms. For instance, Herpes Simplex Virus 1 (HSV-1) encodes the E3 ubiquitin ligase ICP0 that targets PML and induces its proteasomal degradation (Boutell et al., 2003; Wang et al., 2012), whereas the IE1 of human cytomegalovirus (hCMV) disrupts PML NBs by inducing a loss of PML SUMOylation (Schilling et al., 2017).

The antiviral property of PML has been shown in human cells by its overexpression or its depletion. Indeed, expression of specific PML isoforms confers resistance to several DNA and RNA viruses (Everett and Chelbi-Alix, 2007) including HSV-1 (McNally et al., 2008; Cuchet et al., 2011), Varicella-Zoster Virus (VZV) (Reichelt et al., 2011), Adenovirus (Atwan et al., 2016), Human Foamy Virus (HFV) (Regad et al., 2001), Encephalomyocarditis virus (EMCV) (Maroui et al., 2011), Influenza A virus (IAV) (Chelbi-Alix et al., 1998), Vesicular Stomatitis Virus (VSV) (Chelbi-Alix et al., 1998; El Asmi et al., 2014), Dengue virus (DENV) (Giovannoni et al., 2015) or Rabies Virus (Blondel et al., 2010) by interacting with cellular or viral proteins inhibiting their functions (Regad et al., 2001; McNally et al., 2008; Maroui et al., 2011; Reichelt et al., 2011; El Asmi et al., 2014) or to poliovirus in a p53-dependent way by recruiting p53 within PML NBs and inducing apoptosis in infected cells (Pampin et al., 2006). Among all PML isoforms, PMLIV is implicated in innate immunity in addition to its intrinsic antiviral activity (El Asmi et al., 2014). Indeed, modification of PMLIV by SUMO positively regulates virus-induced IFNβ synthesis *via* the recruitment of the peptidylprolyl Cis/Trans Isomerase (PIN1) to PML NBs that results in a higher IRF3 phosphorylation (El Asmi et al., 2014). PML plays key roles in host antiviral defense and mediates the IFN-induced antiviral state since IFN has reduced antiviral activity in the absence of PML (Regad et al., 2001; Everett and Chelbi-Alix, 2007; Geoffroy and Chelbi-Alix, 2011; Maroui et al., 2011).

### PML and SUMO Pathway

PML is subjected to multiple post-translational modifications, which include phosphorylation, acetylation, ubiquitylation, ISGylation, and SUMOylation (Geoffroy and Chelbi-Alix, 2011). PML localization is intimately linked to its SUMOylation. Within the nucleus, most of PML is expressed in the diffuse nuclear fraction of the nucleoplasm with only a small fraction in the matrix-associated NBs (Zhu et al., 1997; Porta et al., 2005; El McHichi et al., 2010). The transfer of PML from the nucleoplasm to PML NBs is associated with PML SUMOylation (Zhu et al., 1997; Muller et al., 1998; El McHichi et al., 2010, El-Asmi et al., 2019).

SUMO is the first identified PML NB-associated protein that interacts with PML (Boddy et al., 1996). SUMOylation has important consequences on PML functions as it can affect its localization, its stability, its ability to interact with other partners and its antiviral property (Everett and Chelbi-Alix, 2007; Geoffroy and Chelbi-Alix, 2011). Also, the non-covalent interactions of SUMO with PML *via* its SUMO-Interacting Motif (SIM) is required for further recruitment of PML NB partners (Shen et al., 2006; van Damme et al., 2010; Maroui et al., 2012). SUMOylation and the SIM in PML contribute substantially to body architecture and to multivalent interactions within PML NBs. In addition, PML exerts a SUMO E3 ligase activity (Chu and Yang, 2011) that facilitates SUMOylation of a large number of critical proteins by concentrating them into PML NBs where it serves as the scaffold.

**FIGURE 2:**
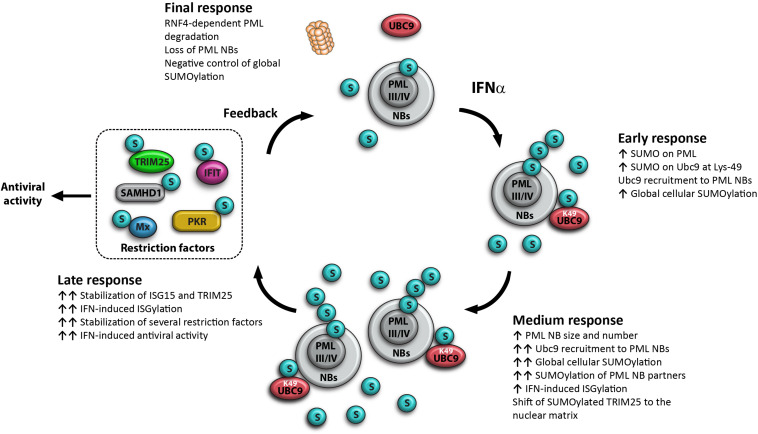
Model for the key role of PML in IFN-increased global cellular SUMOylation. Early response at 45 min post-IFN treatment: IFN increases PML-dependent global cellular SUMOylation, with an enhancement of PML and Ubc9 SUMOylation; IFN induces PML-dependent Ubc9 shift to the nuclear matrix and the recruitment of SUMOylated Lys-49-Ubc9 to PML NBs; PMLIII and PMLIV are key players of the IFN-induced increase of cellular SUMOylation; Medium response at 16 h post-treatment: IFN increases the expression of PML isoforms, resulting in the increase of the number and the size of PML NBs, a further increase of Ubc9 recruitment to PML NBs, a maximum enhancement of global cellular SUMOylation, a positive regulation of IFN-induced ISGylation and a shift of SUMOylated TRIM25 to the nuclear matrix. Late response at 20 h: IFN promotes SUMOylation and a huge stabilization of several restriction factors resulting in enhanced IFN-induced antiviral activity. Final response at 24 h post-IFN treatment: IFN promotes RNF4-mediated proteasomal PML degradation with a loss of PML NBs and a decrease of global cellular SUMOylation, suggesting a negative control of global SUMOylation, this process is essential to ensure a return of the cell to homeostasis.

More recently, it has been shown that IFN enhances rapidly (45 min post-treatment) global cellular SUMOylation in an endogenous PML-dependent manner (Maroui et al., 2018). Also, IFN increases the SUMOylation of PML and of Ubc9, the only known SUMO E2-conjugating enzyme. The SUMOylation of Ubc9 at Lys-49 promotes its recruitment to PML NBs, and is associated with an increase in global SUMOylation within 16 h post-IFN treatment (Maroui et al., 2018) and in enhanced IFN-induced ISGylation (El-Asmi et al., 2020a). This process is followed by the stabilization of several restriction factors and an enhanced IFN-induced antiviral state (El-Asmi et al., 2020a). Later, IFN induces RNF4-dependent PML degradation with a loss of PML NBs (Maarifi et al., 2015) and a decrease of global cellular SUMOylation (Maroui et al., 2018), suggesting a feedback loop for the control of protein SUMOylation (Figure 2).

Interestingly, the expression of each of the human PML isoform (PMLI to PMLIV) into PML−/− cells reveals that increased SUMOylation in response to IFN is orchestrated by PMLIII and PMLIV isoforms (Maroui et al., 2018). The mechanism by which the isoform-specific protein sequences enhance global cellular SUMOylation is still unclear. Although PML isoforms may have related functions due to their common RBCC/TRIM domain, increasing evidences suggest that the variability of the C-terminal region confers specific functions of some PML isoforms (Maroui et al., 2011; Nisole et al., 2013; El Asmi et al., 2014). Importantly, PMLIV acts as a SUMO E3 ligase increasing the SUMOylation of various proteins (Chu and Yang, 2011) and among all PML isoforms, only the expression of PMLIII or PMLIV enhances overall SUMOylation in PML negative cells with a further increase upon IFN treatment (Maroui et al., 2018). As a SUMO ligase, PML interacts with Ubc9 *via* its RING finger motif (Chu and Yang, 2011), and PMLIII or PMLIV promotes the recruitment of SUMOylated Ubc9 within PML NBs. Indeed, the expression of PMLIII or PMLIV in PML−/− cells is able to recruit Ubc9 to PML NBs where both proteins colocalize (Maroui et al., 2018).

It is noteworthy that PML negative cells have a defect in IFN-induced apoptosis (Wang et al., 1998) and in increasing global cellular SUMOylation (Maroui et al., 2018). Many restriction factors and key regulators of IFN pathway are SUMOylated and require this modification for their functions (Hannoun et al., 2016). The expression of PMLIII or PMLIV in PML negative cells induces IFN-enhanced global cellular SUMOylation and could therefore restore IFN functions.

## The SUMO, Ubiquitin, ISG15 Pathways and Protein Stability

In addition to ubiquitin, several Ubls are expressed following IFN stimulation, including SUMO and ISG15, which are key players of cellular antiviral defense (Hannoun et al., 2016; Maarifi et al., 2016; Dzimianski et al., 2019; El-Asmi et al., 2020b). Like ubiquitin, these Ubls can be conjugated to protein substrates *via* their own E1-E2-E3 enzymatic cascades. Ubc9, and UbcH8 are specific E2 conjugating enzymes for SUMOylation and ISGylation, respectively (Desterro et al., 1997; Johnson and Blobel, 1997; Kim et al., 2004). In some cases, a single Ubl is conjugated to a lysine residue while in other a long chain of polymerized Ubls is attached to an acceptor lysine. Ubiquitin and Ubls are involved in the regulation of a variety of cellular activities, including signal transduction, protein stability, intracellular trafficking and antiviral defense.

Several key players of IFN response are targets of ISGylation (Zhao et al., 2005) and/or SUMOylation (Maroui et al., 2018; El-Asmi et al., 2020a), and have been identified with different experimental global or specific approaches including affinity purification combined with MS. Many proteins implicated in IFN-induced antiviral defense can be targeted by both ISG15 and SUMO namely STAT1, RIG-I, IRF3, PKR, PML, IFIT1, MxA, TRIM25, p53 (Giannakopoulos et al., 2005; Zhao et al., 2005; Maroui et al., 2018; Perng and Lenschow, 2018; El-Asmi et al., 2020a). The consequences of ISGylation and SUMOylation on their functions are summarized in Table 1.

**TABLE 1:** Effects of ISGylation and SUMOylation on key regulators of IFN pathway.

Key regulators of IFN pathway	ISGylation	SUMOylation	References
**IFN production**			
IRF3	Regulates positively IRF3 activation	Reduces virus-induced IFN synthesis	Kubota et al., 2008; Shi et al., 2010
RIG-I	Reduces levels of virus-induced IFN promoter activity	Enhances type I IFN production	Kim et al., 2008; Mi et al., 2010
MDA5	Required for IFN synthesis	Regulates positively IFN synthesis	Fu et al., 2011; Kim et al., 2016; Liu et al., 2020
**IFN signaling**			
STAT1	Preserves STAT1 phosphorylation	Reduces STAT1 phosphorylation	Malakhov et al., 2003; Ungureanu et al., 2005; Maarifi et al., 2015
**Restriction factors**			
MxA	The significance has not been evaluated	Increases protein stabilization and antiviral property	Zhao et al., 2005; Maarifi et al., 2016
PML/TRIM19*	Enhances STAT1 association with PML NBs	Required for PML NB formation and functions Required for antiviral property	Everett and Chelbi-Alix, 2007; Shah et al., 2008; Geoffroy and Chelbi-Alix, 2011; Fan et al., 2020
P53*	Promotes p53 degradation	Promotes cellular senescence	Zhao et al., 2005; Marcos-Villar et al., 2013; Huang et al., 2014
IFIT1	The significance has not been evaluated	Upregulates its protein level in response to IFN	Zhao et al., 2005; El-Asmi et al., 2020a
SAMHD1	The significance has not been evaluated	Upregulates its protein level in response to IFN	El-Asmi et al., 2020a
PKR	Promotes PKR eIF2a activation in the absence of viral infection	Promotes PKR eIF2a activation in the absence of viral infection	Okumura et al., 2013; de la Cruz-Herrera et al., 2014; Maarifi et al., 2018
**Ubl modifiers**			
Ubiquitin	Negatively regulates turnover of ubiquitinated proteins	Promotes Ubc9 localization within PML NBs	Fan et al., 2015; Maroui et al., 2018; McManus et al., 2018
ISG15		Poly-SUMOylation enhances IFN-induced ISG15 and ISGylation	El-Asmi et al., 2020a
TRIM25 (E3 ligase)	Inhibits its own ISGylase activity	Upregulates its protein level in response to IFN	Zou et al., 2007; El-Asmi et al., 2020a
HERC5 (E3)	HERC5 is itself a target for ISGylation, but its functional significance is unknown.	Upregulates its protein level in response to IFN	Wong et al., 2006; El-Asmi et al., 2020a

*All the keys regulators of IFN pathway indicated in the table are highly stabilized by SUMO3 in response to IFN except PML and p53, which are downregulated. PML and p53 are SUMOylated 1 h post IFN stimulation. PML recruits SUMOylated Ubc9 within PML NBs and promotes IFN-enhanced global SUMOylation. Later during IFN treatment PML is degraded suggesting a negative control of SUMOylation.

### Interplay Between Poly-SUMO Chains and Ubiquitin Resulting in Protein Degradation

#### IFN Enhances the SUMOylation of Ubiquitin and Ubiquitin-Like Modifiers

The global identification of proteins conjugated to SUMO in response to IFNα has been performed using a novel proteomics approach based on SUMO remnant immunoaffinity purification (Galisson et al., 2011; Lamoliatte et al., 2014; Lamoliatte et al., 2017; Maroui et al., 2018; El-Asmi et al., 2020a). SUMO proteome analysis revealed that various SUMOylation sites are increased in response to IFNα in ISG products including several restriction factors (e.g., PML, ADAR1, Vimentin, SAMHD1, PKR, IFIT1, IRF1, and ISG20) and other regulators of IFN signaling or IFN production (e.g., STAT1, IRF1, IRF9, Tif1α/TRIM24, TRIM28, TRIM33, and TBK1) (Maroui et al., 2018; El-Asmi et al., 2020a). In addition to the three major SUMO sites on Lys-65, Lys-160 and Lys-490 (Kamitani et al., 1998), several other SUMO sites have been more recently identified on PML (Lys-209, Lys-226, Lys-337, Lys-380, Lys-394, Lys-400, Lys-401, Lys-476, Lys-478, Lys-487, Lys-497, and Lys-616) (El-Asmi et al., 2020a), but only Lys-65, Lys-380, and Lys-490 showed more than fourfold increase in SUMOylation upon IFNα stimulation (Maroui et al., 2018). Interestingly, most of the proteins from the SUMOylation machinery (e.g., Ubc9, SUMO1, SUMO2, SUMO3, PIAS1, PIAS2, RanBP2, TRIM28, and PML) display increased SUMOylation in response to IFNα (Maroui et al., 2018).

#### Recruitment of SUMOylated Ubc9 on PML NBs in Response to IFN

Ubc9 is SUMOylated at Lys-14, Lys-18, Lys-48, Lys-49, Lys-65, and Lys-154 and its SUMOylation at Lys-14 was shown to display enhanced binding to SIM-containing proteins (Knipscheer et al., 2008; Wieczorek et al., 2016; Maroui et al., 2018). Only SUMOylation at Lys-49 of Ubc9 is enhanced in the nucleus at an early time post-IFNα treatment (45 min), and further increase of SUMOylation at Lys-49 is found in the cytoplasm and the nucleus during prolonged IFNα treatment (Maroui et al., 2018). In untreated cells, Ubc9 is expressed in the cytoplasm and the nucleoplasm. Remarkably, IFNα induces PML-dependent Ubc9 transfer to the nuclear matrix where Ubc9 colocalizes with PML within PML NBs resulting in an enhancement of global cellular SUMOylation (Maroui et al., 2018). The SUMOylation of Ubc9 at Lys-49 promotes its localization to PML NBs (McManus et al., 2018). Altogether, these findings demonstrate that SUMOylated Ubc9 and PML are key players of IFN-enhanced global cellular SUMOylation.

#### Interplay Between Poly-SUMO Chains and Ubiquitin Resulting in Degradation of ISG Products

SUMOylation of proteins can alter their interaction properties and thereby their localization and/or stability thus providing a rapid and reversible way to control protein functions. SUMO is conjugated to lysine residues in target proteins through an isopeptide linkage catalyzed by SUMO-specific activating (E1), conjugating (E2), and ligating (E3) enzymes. Due to the expression of several SUMO paralogs, SUMO pathway analysis is more complicated than that of ubiquitin or ISG15. Mammalian cells can express four SUMO paralogs with SUMO1, SUMO2, and SUMO3 being conjugatable (SUMOylation) and ubiquitously expressed, while SUMO4 expression is restricted to renal, immune and pancreatic cells (Flotho and Melchior, 2013; Hay, 2013; Baczyk et al., 2017). Also, SUMO4 contains a proline residue close to the diglycine motif, which prevents its maturation and conjugation (Owerbach et al., 2005). In addition, SUMO *via* the SIMs is able to effect protein activity without being covalently attached (Song et al., 2004).

SUMO1 and SUMO2/3 (SUMO2 and SUMO3 have 97% peptide sequence similarity and are collectively referred to as SUMO2/3) modify both common and different substrates and growing evidences show that they may have distinct functions (Lallemand-Breitenbach et al., 2008; Tatham et al., 2008; Flotho and Melchior, 2013; Maarifi et al., 2015; Maarifi et al., 2018). SUMO2 and SUMO3 contain a lysine residue at position 11 (Lys-11) that can be used for self-conjugation and that is usually the site of poly-SUMO chains. In contrast, SUMO1 does not contain Lys-11 and therefore does not form poly-SUMO chains. However, alternatively linked non-canonical SUMO2/3 chains, mixed SUMO1-SUMO2/3 chains and SUMO2/3 chains capped with SUMO2, have been described (Sriramachandran and Dohmen, 2014; Gartner et al., 2018; Sriramachandran et al., 2019). It is well established that SUMO2/3 have the capability to efficiently form highly branched poly-SUMO chains that have the ability to recruit SUMO-targeted ubiquitin ligases (STUbls) such as the RING finger protein RNF4 and RNF111 (Sriramachandran and Dohmen, 2014; Kumar and Sabapathy, 2019). RNF4 harbors multiple SIMs in its N-terminus region that allow a strong interaction with SUMO leading to the ubiquitylation of poly-SUMO chains conjugated to substrates and resulting in the proteasomal degradation of SUMO2/3 conjugated proteins (Hay, 2013). The best-studied case is the SUMO-dependent PML degradation in cells treated with arsenic trioxide (As_2_O_3_) (Lallemand-Breitenbach et al., 2008; Tatham et al., 2008; Erker et al., 2013) or with IFN (Maarifi et al., 2015). More recently, RNF111 was reported to select proteins carrying SUMO1-capped SUMO2/3 hybrid conjugated and targets them for proteasomal degradation (Sriramachandran et al., 2019).

Treatment with IFNα results in a very rapid increase of global cellular SUMOylation (Maroui et al., 2018), which also increase the SUMOylation of endogenous PML and Sp100. This is followed by the increase of their mRNA and protein expression, and by their RNF4-dependent proteasomal degradation during prolonged IFN treatment (Maarifi et al., 2015). It should be noted that Sp100 is an ISG product permanently associated to PML NBs (Grotzinger et al., 1996). Therefore, the RNF4-dependent proteasomal degradation of PML and Sp100 proteins results in a loss of PML NBs. The expression of the restriction factors TRIM5α, p53 and Daxx is also dowregulated by SUMO3 in response to IFNα (El-Asmi et al., 2020a). Large-scale proteomic analyses revealed that in response to IFNα, SUMO3 expression results in the down-regulation of 586 proteins such as the restriction factor IFI16, which expression is restored upon RNF4 depletion (El-Asmi et al., 2020a) as previously shown for PML and Sp100 (Maarifi et al., 2015). The SUMOylation of PML and Sp100 is required for their degradation, suggesting that SUMOylation of other ISG products could also be necessary for their dowregulation.

### SUMO and ISG15 Pathway in Response to IFN

#### IFN Stabilizes ISG15 and ISG15 Modifiers

ISG15 is expressed as a 17 kDa precursor protein that is processed into its mature 15 kDa form *via* protease cleavage to expose a carboxy-terminal LRLRGG motif (Potter et al., 1999). ISG15 exists as an unconjugated protein and is also covalently conjugated to lysine residues of several substrates through this motif by a process known as ISGylation (Loeb and Haas, 1992; Narasimhan et al., 1996). Similar to ubiquitin and SUMO, ISG15 can non-covalently bind to proteins and modulate their functions (Perng and Lenschow, 2018).

ISG15 was first identified from the study of type I IFN-treated cells (Korant et al., 1984; Haas et al., 1987). Whereas some Ubls are constitutively expressed in the host cell, ISG15 and the members of the enzymatic cascade mediating ISG15 conjugation, including the E1, E2, E3, and the deconjugating enzyme, are ISG products themselves (Loeb and Haas, 1992), suggesting their tight link to IFN-regulated cellular functions. The expression of the *ISG15* gene is dependent on an ISRE found in its promoter region (Reich et al., 1987). The increase level of free ISG15 occurs within 2 h post-IFN treatment and is maximal by about 18 h, whereas increase of ISGylation is observable at least 12h post-IFN treatment. In addition, ISG15 is also induced by viral and bacterial infections (Yuan and Krug, 2001; Radoshevich et al., 2015), lipopolysaccharide (LPS) (Malakhova et al., 2002) and retinoic acid (Pitha-Rowe et al., 2004). Using MS, hundreds of host proteins have been identified as ISG15 targets upon IFN stimulation (Giannakopoulos et al., 2005; Zhao et al., 2005). Among these, the antiviral effectors, STAT1, IRF3, Retinoic acid Inducible Gene (RIG-I), double-stranded RNA (dsRNA)-dependent protein kinase (PKR), IFN-induced protein with tetratricopeptide repeats 1 (IFIT1), p53 and MxA (Giannakopoulos et al., 2005; Zhao et al., 2005; El-Asmi et al., 2019) (Table 2). Remarkably, the SUMO E3 ligase polycomb 2 (Pc2) and the SUMO protease SENP1 were also identified as ISG15 targets (Zhao et al., 2005), suggesting a possible crosstalk between the ISG15 and SUMO pathways. For a subset of these potential targets, modification has been validated, and the impact of ISGylation on their function has been investigated (Perng and Lenschow, 2018) (Table 1).

**TABLE 2:** Antiviral properties of the restriction factors stabilized by SUMO3 in the presence of IFNα.

Protein stability	SUMO3/wt cells	(SUMO3/wt cells) + IFN	Viral resistance
**IFN signaling**			
STAT1	+	+++	
STAT2		+++	
IRF9		+++	
**Restriction factors**			
MxA	+	+++	VSV, IAV, VACV
MxB		+++	HIV-1, HSV-1, HSV-2
GBP1		+++	VSV, IAV, DENV, KSHV
GBP5		+++	IAV, RSV, HIV-1
SAMHD1	+	+++	HIV-1, HSV-1, HBV, VACV
IFITM1		+++	ZIKV, DENV
IFITM2		+++	MLV, SARS-CoV-2
IFITM3		+++	MLV,VSV, IAV, IBV, DENV, SARS-CoV-2
IFIT1	+	+++	HBV, HPV, HCV
IFIT2		+++	VSV
IFIT3		+++	HBV, HPV, HCV
IFI44		+++	HIV-1, RSV
IFI44L		+++	HCV, RSV
TRIM21		+++	CVB3, HBV
Tetherin/BST2		+++	HIV-1, VSV, KSHV
PKR	+	+++	EMCV, VSV
ISG15		+++	IAV, IBV, Sindbis virus

Unlike ubiquitylation, ISGylation does not appear to directly target proteins for proteasome-mediated degradation (Liu et al., 2003). It has been reported that ISG15 can compete with ubiquitin for ubiquitin binding sites on a protein, thereby indirectly impairing the ubiquitin/proteasome pathway by interfering with protein degradation (Desai et al., 2006). In addition, ISG15-ubiquitin mixed chains have been identified and may negatively regulate the turnover of ubiquitylated proteins (Fan et al., 2015).

In response to IFN, SUMO3 expression results in a stabilization of ISG15 E2 conjugating enzyme Ube2L6/UbcH8 and the E3 ISG15 ligases TRIM25 and HERC5 (HECT domain and RCC1-like domain containing protein 5) (El-Asmi et al., 2020a) highlighting an unsuspected interplay between SUMOylation and ISGylation. Importantly, the upregulation of ISGylation in response to IFNα is observed in SUMO3- but not in SUMO1-expressing cells (El-Asmi et al., 2020a), thus providing a novel differential effect of SUMO1 and SUMO3 on the positive regulation of ISG products and IFNα-induced ISGylation.

Furthermore, Ni-NTA analysis revealed that IFNα drastically enhances global cellular ISGylation and ubiquitylation in cells overexpressing His-SUMO3, thus demonstrating that ISG15 may modify SUMO2/3 conjugated proteins (El-Asmi et al., 2020a) as previously shown for ubiquitin (Tatham et al., 2008). The crosstalk between SUMOylation and ISGylation is further demonstrated by Ubc9 depletion that results in a decrease of cellular SUMOylation and a decrease of IFN-induced cellular ISGylation (El-Asmi et al., 2020a).

#### TRIM25 a Key Player of ISG15 Modifiers

Several members of the tripartite motif-containing (TRIM) superfamily are expressed in response to IFNs and are implicated in various biological processes associated with innate immunity (Rajsbaum et al., 2008). This superfamily contains in its N-terminal region a TRIM (also named RBCC) motif that comprises a RING finger, one or two B-box domains and a Coiled-Coil domain in the amino-terminal region (Reymond et al., 2001; Ozato et al., 2008). The presence of a RING finger, which can mediate the conjugation of proteins with ubiquitin, SUMO or ISG15, contributes to the biological responses of TRIM proteins (Ozato et al., 2008).

TRIM25 (also named EFP, RNF147, and ZNF147) is an IFN-inducible E3 ligase by type I and type II IFNs (Nakasato et al., 2006; Martin-Vicente et al., 2017). TRIM25 participates in multiple cellular processes including the regulation of the antiviral innate immunity (Manokaran et al., 2015; Martin-Vicente et al., 2017; Chen et al., 2019; Lin et al., 2019). TRIM25 is involved in the RIG-I-mediated antiviral response by inducing the Lys-63-linked poly-ubiquitylation of RIG-I, and consequently the induction of type I IFN production (Gack et al., 2007). The importance of TRIM25 in RIG-I signaling was illustrated by reduced IFN production in TRIM25−/− mouse embryonic fibroblasts and a corresponding susceptibility to viral infection (Gack et al., 2007). In contrast, TRIM25 expression inhibits late-stage replication of HIV-1 and Murine Leukemia Virus (MLV) (Uchil et al., 2008). Remarkably, nuclear TRIM25 protein confers resistance to IAV independently of both its ubiquitin ligase activity and the IFN induction pathway (Meyerson et al., 2017). TRIM25 directly restricts viral RNA synthesis in infected cells by binding to nuclear viral ribonucleoproteins, demonstrating that TRIM25 exerts an intrinsic antiviral activity by interacting with a viral protein inhibiting its function.

TRIM25 acts as an E3 ligase that can catalyze both ubiquitylation and ISGylation (Nakasato et al., 2006; Zou and Zhang, 2006). In addition, increased expression of TRIM25 enhances ubiquitylation of multiple cellular proteins (Zheng et al., 2017). Also, TRIM25 can undergo auto ISGylation on Lys-117 residue, a modification that negatively regulates its ISG15 E3 ligase activity (Nakasato et al., 2006; Zou et al., 2007). In addition to be ISGylated and ubiquitylated (Nakasato et al., 2006), TRIM25 has been shown to be SUMOylated by Ni-NTA purification analysis, with a further increase of its modification upon IFN stimulation (El-Asmi et al., 2020a). However, the SUMO site of TRIM25 has not yet been identified.

The unmodified form of TRIM25 is found in the RIPA soluble fraction containing the cytoplasm and the nucleoplasm. Remarkably, IFNα enhances TRIM25 SUMOylation and shifts SUMO-modified TRIM25 to the nuclear matrix, which also contains most of the SUMOylated proteins and a small portion of ISGylated proteins (El-Asmi et al., 2020a). Whether SUMOylated TRIM25 is recruited within PML NBs colocalizing with PML is still unknown.

The E3 ISG15 ligases TRIM25 and HERC5 are stabilized by SUMO3 in IFNα-treated cells and their depletion expectedly impairs protein ISGylation, but only depletion of TRIM25 abrogates the stabilization of ISG products (El-Asmi et al., 2020a), demonstrating that TRIM25 is a key player in the SUMO3-mediated protein stabilization in response to IFN. Further experiments are required to determine the precise function of TRIM25 in this process.

Remarkably some members of TRIM protein family act as E3 ligases that can catalyze ubiquitylation, ISGylation and SUMOylation (Meroni and Diez-Roux, 2005; Nakasato et al., 2006; Zou and Zhang, 2006; Gillot et al., 2009; Chu and Yang, 2011). As seen for TRIM25, a single TRIM protein can have dual E3 activities. It is now of great interest to determine the regulation of the TRIM proteins’ SUMO E3 activity, its targets, and its interplay with the ubiquitin and ISG15 E3 activities.

#### Interplay Between Poly-SUMOylation and ISG15 Resulting in Protein Stabilization

SUMO3 but not SUMO1 stabilizes several ISG products in response to IFN. The large-scale proteomic analyses revealed that SUMO3 stabilizes 583 proteins in response to IFNα, among which are several proteins implicated in IFN production such as MAVS (also known as IPS-1/VISA/Cardif), TBK1, IRF3, RIG-I, and MDAR, in IFN signaling (STAT1, STAT2, and IRF9) and in antiviral defense such as SAMHD1, Tetherin/BST2, PKR, OAS3, SAMD9, TRIM21, ISG15 and members of GBP, IFITM, IFIT and IFI families (El-Asmi et al., 2020a).

The upregulation by SUMO3 of key players of IFN signaling STAT1, STAT2 and IRF9 in response to IFN (Table 2) could enhance ISG products independently of their SUMOylation since constitutive expression of ISGs can be mediated by the unphosphorylated ISGF3 (U-ISGF3) complex, consisting of IRF9 together with unphosphorylated STAT1 and STAT2 (Cheon et al., 2013; Sung et al., 2015; Wang et al., 2017b). While the transcription factor ISGF3 (IRF9 and tyrosine-phosphorylated STAT1 and STAT2) drives the first rapid response phase post-IFN treatment, the stabilization by SUMO3 of the U-ISGF3 (El-Asmi et al., 2020a) could drives a second prolonged response of ISG increase.

Ni-NTA analysis revealed that IFNα drastically enhances global cellular ISGylation in cells expressing His-SUMO3 (El-Asmi et al., 2020a), demonstrating that ISG15 may modify SUMO2/3 conjugated proteins as previously shown for ubiquitin (Tatham et al., 2008). In contrast, Ubc9 depletion decreases cellular SUMOylation and remarkably results in a decrease of global cellular ISGylation (El-Asmi et al., 2020a), further demonstrating the crosstalk between SUMOylation and ISGylation.

Significant progress has been made to understand the functional significance of protein ISGylation. Unlike ubiquitylation, ISGylation seems to counteract proteasome-dependent protein degradation *via* the conjugation of ISG15 to different E2 and E3 ubiquitin-conjugating enzymes (Okumura et al., 2008) or *via* the formation of mixed ubiquitin-ISG15 chains (Fan et al., 2015), thus resulting in a decrease of poly-ubiquitylated protein levels and protein protection from proteasomal degradation. Remarkably, many of the ISG products stabilized by SUMO3 in response to IFN (El-Asmi et al., 2020a) have been previously shown to be ISGylated such as MxA, GBP1, SAMHD1, PKR, IFIT1, IFIT3, UBE2L6, and HERC5 (Giannakopoulos et al., 2005; Zhao et al., 2005) (Table 1). The recent findings that the formation of the poly-SUMO chains results in enhanced IFNα-induced global ISGylation, and in a TRIM25-dependent stabilization of several ISG products highlight the consequences of the crosstalk between SUMOylation and ISGylation on protein stabilization. Further experiments are needed to identify SUMO-ISG15 mixed chains and to determine whether TRIM25 could act as a SUMO-ISG15 ligase.

## SUMO3 Enhances IFN-Induced Antiviral Activities by Stabilizing Several Restriction Factors

Several restriction factors playing key roles in antiviral defense are stabilized by SUMO3 in response to IFN (El-Asmi et al., 2020a), they include SAMHD1, Tetherin/BST2, PKR, oligoadenylate synthetase (OAS)3, ISG15 and members of Mx, GBP, IFITM, IFIT, and IFI families (Al-Khatib et al., 2003; Goldstone et al., 2011; Hollenbaugh et al., 2013; Kane et al., 2013; Kim et al., 2013; Goujon et al., 2014; Krapp et al., 2016; Crameri et al., 2018). Several of these restriction factors are known to confer viral resistance by targeting different steps of viral replication (Blondel et al., 2015; El Asmi et al., 2018). For example, IFITM proteins inhibit entry (Weidner et al., 2010), MxA inhibits primary transcription (Staeheli and Pavlovic, 1991), IFIT, PKR and OAS inhibit viral translation (Balachandran et al., 2000; Terenzi et al., 2006; Kristiansen et al., 2011), and Tetherin prevents release of virions from the cell (Weidner et al., 2010).

Importantly, the increase in abundance of these restriction factors by SUMO3 in IFNα-treated cells is correlated with the increase of IFNα-induced anti-HIV-1 and anti-HSV-1 activities (El-Asmi et al., 2020a). In contrast, the loss of SUMOylation by Ubc9 depletion reduces the IFNα-mediated resistance to these viruses (Sahin et al., 2014). Taken together, these results demonstrate that the stabilization of these restriction factors by SUMO3 results in enhanced IFNα-induced antiviral defense. The antiviral activity of the most studied of these restriction factors is briefly described below and conveniently summarized in Table 2.

### Mx Proteins (MxA and MxB)

Myxovirus resistance (Mx) proteins are known for their protection of mice from infection by IAV (Horisberger et al., 1983; Staeheli et al., 1986). They belong, like Guanylate Binging Proteins (GBP), to the guanosine triphosphatase (GTPase) subfamily. Humans have two *Mx* genes: *MxA* and *MxB*, which expression is strictly controlled by type I and type III IFNs. MxB shares 63% amino acid sequence identity with MxA. MxA (also known as MX1) accumulates in the cytoplasm and is partly associated with the plasma membrane as well as the smooth endoplasmic reticulum (Accola et al., 2002; Stertz et al., 2006). MxB (also known as MX2) exists in two isoforms, a long 78 kDa and a short 76 kDa molecule (Melen et al., 1996). The long 78 kDa MxB has a nuclear localization sequence (NLS) and is localized near the nuclear pores, whereas the 76 kDa isoform lacking the NLS is cytoplasmic.

MxA is SUMOylated at Lys-48 and interacts non-covalently with SUMO *via* two SIMs located in the GTPase binding domain that are required for its antiviral activity (Brantis-de-Carvalho et al., 2015). MxA is stabilized through its oligomerization in SUMO-expressing cells (Maarifi et al., 2016). MxA oligomerization capacity is important for its GTPase activity, and also for its interaction with SUMO and Ubc9 (Brantis-de-Carvalho et al., 2015). Accordingly, monomeric mutant MxA-L612K has a reduced interaction with SUMO and Ubc9 (Brantis-de-Carvalho et al., 2015), and is rapidly degraded in cells compared to wild-type MxA, demonstrating that the self-assembly of the MxA protein is critical for protein stability (Janzen et al., 2000).

Interestingly, the overexpression of each SUMO paralog confers resistance to VSV through MxA stabilization and MxA depletion in SUMO-expressing cells abrogates the anti-VSV effect of SUMO, thus signifying that MxA is a key mediator of SUMO-induced resistance to VSV (Maarifi et al., 2016). Although IFN production is highly reduced in VSV-infected SUMO-expressing cells, the intrinsic anti-VSV activity of SUMO mediated by MxA is still maintained. Further experiments are required to determine whether the replication process of other viral families can be inhibited by SUMO expression *via* the stabilization of MxA or other restriction factors. All SUMO paralogs stabilize MxA protein, however, SUMO3, but not SUMO1, promotes a higher increase of MxA protein level upon IFN stimulation compared to wild-type cells (El-Asmi et al., 2020a).

MxA inhibits a wide variety of RNA and DNA viruses. The overexpression of MxA protein inhibits the multiplication of several RNA viruses such as IAV, VSV, measles virus, and other viruses belonging to the family Bunyaviridae (Haller et al., 2007). MxA protein also confers resistance to a few DNA viruses like the vaccinia virus (VACV), the monkey poxvirus, and the African swine fever virus (Mitchell et al., 2013). In contrast to MxA, the level of MxB protein is not affected by SUMO alone, though SUMO3 promotes a significant increase in MxB protein level in response to IFNα compared to wild-type cells (El-Asmi et al., 2020a).

MxB was long considered non-antiviral, until the finding in 2013 that human immunodeficiency virus type 1 (HIV-1) is inhibited by MxB (Goujon et al., 2013; Kane et al., 2013; Liu et al., 2013). Since then, MxB was shown to restrict several members of the Flaviviridae, including Hepatitis C Virus (HCV), Japanese encephalitis virus and DENV (Yi et al., 2019) as well as several herpesviruses, including HSV-1, HSV-2, and Kaposi’s sarcoma-associated herpesvirus (KSHV) (Crameri et al., 2018). MxB restriction of HSV-1 and HSV-2 requires GTPase function, in contrast to restriction of lentiviruses.

Nuclear MxB was found by Ni-NTA analysis to be conjugated to SUMO3 with enhanced modification upon cell treatment with IFNα (El-Asmi et al., 2020a). Further investigations are needed to identify the MxB SUMO sites and to determine whether MxB SUMOylation is required for its antiviral activity. Both MxA and MxB proteins are stabilized by SUMO3 in response to IFNα (El-Asmi et al., 2020a) (Table 2), therefore their upregulation could contribute to the enhanced IFN-induced antiviral activity.

### GBP Proteins (GBP1 and GBP5)

Guanylate-Binding Proteins (GBP) are a family of dynamin-related large GTPases, which are expressed in response to IFNs (Vestal and Jeyaratnam, 2011; Pilla-Moffett et al., 2016). Currently, 7 human GBPs have been identified. Recently, proteomic analysis revealed that SUMO3 upregulates the expression of the antiviral proteins GBP1 and GBP5 in response to IFNα (El-Asmi et al., 2020a).

GBP1 is abundantly expressed during the innate immune response (Vestal and Jeyaratnam, 2011; Pilla-Moffett et al., 2016). It is one of the large GTPases, with a relative molecular mass of 67 kDa, which can hydrolyze GTP to GDP and subsequently to GMP (Prakash et al., 2000). It is a large self-activating GTPase, and its dimerization is necessary for sufficient GTP-hydrolyzing activity (Chappie et al., 2010). The GTPase activity of GBP1 is required for its antiviral activity (Vestal, 2005). GBP1 exhibits antiviral activity against various RNA viruses such as VSV, DENV, IAV, classical swine fever virus, and HCV (Anderson et al., 1999; Itsui et al., 2009; Pan et al., 2012; Li et al., 2016). In addition, overexpression of GBP1 significantly inhibits Kaposi’s sarcoma-associated herpesvirus (KSHV) infection, while the knockdown of GBP1 promotes KSHV infection. The GTPase activity and dimerization of GBP1 are responsible for its anti-KSHV activity (Zou et al., 2017).

GBP5 overexpression confers resistance to IAV by increasing the expression of virus-induced IFN and ISGs, while knockdown of GBP5 has the opposite effect (Feng et al., 2017). Also, GBP5 inhibits respiratory syncytial virus (RSV) infection by reducing the cell-associated levels of the RSV small hydrophobic protein, which is a viroporin (Li et al., 2020) and reduces HIV-1 infectivity by interfering with maturation and virion incorporation of the envelope glycoprotein (Krapp et al., 2016; Hotter et al., 2017). However, whether GBP1 and GBP5 are SUMOylated and whether their modifications are required for their stability and/or antiviral property are still unknown.

### SAMHD1

Sterile-α-motif and HD domain containing protein 1 (SAMHD1) regulates the cellular dNTP (2′-deoxynucleoside-5′-triphosphate) pool by catalyzing the hydrolysis of dNTP into 2′-deoxynucleoside and triphosphate products. By limiting the supply of dNTPs for viral DNA synthesis, SAMHD1 restricts the replication of several retroviruses, such as HIV-1, and some DNA viruses, vaccinia virus (VACV) and HSV-1, in dendritic and myeloid lineage cells and resting T-cells (Baldauf et al., 2012; Lahouassa et al., 2012; Gramberg et al., 2013; Hollenbaugh et al., 2013; Sze et al., 2013; Chen et al., 2014). SAMHD1 is SUMOylated at several lysine sites (Lys-43, Lys-148, Lys-294, Lys-304, Lys-332, Lys-467, Lys-469, Lys-492, Lys-523, Lys-544, Lys-595, and Lys-622) but only SUMOylation at the SAMHD1 consensus site of Lys-622 is enhanced in response to IFNα (El-Asmi et al., 2020a).

SAMHD1 protein is stabilized by SUMO3 alone with a much higher stabilization upon IFNα stimulation compared to wild-type cells (El-Asmi et al., 2020a). Also, it is interesting to note that SUMOylated SAMHD1 and MxB interact together in response to IFNα, suggesting that under certain conditions SUMOylated MxB and SAMHD1 can cooperate to confer viral resistance in IFN-treated cells. However, it is still unknown whether the SUMOylation of SAMHD1 is required for its antiviral activity.

### Tetherin/BST2

Tetherin also know as bone marrow stromal antigen 2 (BST-2) is induced by type I IFN (Neil et al., 2008) and its protein level is stabilized by SUMO3 in response to this cytokine (El-Asmi et al., 2020a). The antiviral property of tetherin was demonstrated by its capacity to restrict the release of virions from HIV-1-infected cells (Neil et al., 2008). Since then, tetherin has been shown to potently block viral replication by inhibiting enveloped virus budding from the surface of infected cells (Swiecki et al., 2013; Berry et al., 2018). Tetherin restricts viruses from different families: arenaviruses (Lassa and Machupo) (Sakuma et al., 2009); herpesviruses (KSHV) (Mansouri et al., 2009); filoviruses (Ebola and Marburg) (Jouvenet et al., 2009; Sakuma et al., 2009); rhabdoviruses (VSV) (Weidner et al., 2010); paramyxoviruses (Nipah) (Radoshitzky et al., 2010) and flaviviruses (HCV) (Dafa-Berger et al., 2012).

### IFITM Proteins (IFITM1, IFITM2, and IFITM3)

Humans have three IFN-induced transmembrane proteins (IFITM1, IFITM2, and IFITM3) (Siegrist et al., 2011). IFITM proteins are involved in the regulation of many activities, such as immune signal transduction and antiviral defense. It has been shown recently that the protein expression of these three IFITM members is stabilized by SUMO3 in response to IFNα (El-Asmi et al., 2020a).

IFITM proteins have been reported to suppress the entry of a wide range of viruses (Amini-Bavil-Olyaee et al., 2013), though other inhibitory mechanisms affecting entry have been proposed (Brass et al., 2009; Huang et al., 2011; Lu et al., 2011; Yu et al., 2015). IFITM1, IFITM2, and IFITM3 potently inhibit HIV-1 replication at least partially through interfering with virus entry (Lu et al., 2011). More recently, a novel mechanism by which IFITM proteins inhibit viral infection has been described. Indeed, IFITMs might also inhibit viral gene expression and viral protein synthesis (Lee et al., 2018; Liao et al., 2019). In addition, IFITM1/2/3 proteins confer resistance to different other viruses including IAV (Feeley et al., 2011), West Nile Virus (WNV), DENV (Jiang et al., 2010), VSV (Weidner et al., 2010), SARS Coronavirus (SARS-CoV), and Marburg virus (MARV) (Huang et al., 2011). The precise inhibitory mechanism of IFITMs on viral infection and replication still requires further exploration but their elevated expression by SUMO3 in response to IFN could enhance their antiviral response.

### IFIT Proteins (IFT1, IFT2, and IFIT3)

IFN-induced proteins with tetratricopeptide repeats (IFITs) motifs play important roles in host innate immune response to viruses. There are four members in human IFIT family: IFIT1 (ISG56), IFIT2 (ISG54), IFIT3 (ISG60 or IFIT4) and IFIT5 (ISG58). The promoters of their genes, which are clustered, contain the ISREs and are induced by type I IFNs. IFIT1, IFIT2, and IFIT3 are stabilized by SUMO3 in response to IFNα with an increase of SUMOylation on four sites of IFIT1 (Lys-199, Lys-336, Lys-370, and Lys-407) (El-Asmi et al., 2020a).

IFITs are cytoplasmic proteins and do not have any known enzymatic roles. However, they are implicated in cellular functions by mediating protein–protein interactions and forming multiprotein complexes with cellular and viral proteins through their multiple tetratricopeptide repeats motifs (D’Andrea and Regan, 2003). IFIT1 and IFIT2 directly interact with eIF3, thus resulting in an inhibition of protein synthesis (Guo et al., 2000).

The IFIT family, especially IFIT1 and IFIT3, restrict DNA and RNA virus replication, such as hepatitis B virus (HBV), human papillomavirus (HPV), HCV, West Nile virus (WNV) (Saikia et al., 2010; Pichlmair et al., 2011; Raychoudhuri et al., 2011; Zhou et al., 2013). In addition, IFIT2 may limit replication of VSV in brain (Fensterl et al., 2014).

### IFI Proteins

IFI44 has 444 amino acids, whereas IFI44L has 452 residues; the two proteins share 45% amino acid identity. Recently, it has been shown that the protein abundance of both IFI44 and IFI44L is enhanced by SUMO3 in response to IFNα (El-Asmi et al., 2020a). Overexpression of IFI44 has been shown to restrict Bunyamwera virus (Carlton-Smith and Elliott, 2012) and HIV-1 (Power et al., 2015) infection. IFI44L has weak antiviral activity against HCV infection (Schoggins et al., 2011). Recently, it has been reported that both IFI44 and IFI44L restrict replication of RSV (Busse et al., 2020). Indeed, overexpression of IFI44 or IFI44L is sufficient to restrict RSV infection at an early time post-infection. Knocking out these genes in human cells increases levels of infection, thus demonstrating a function for IFI44 and IFI44L in controlling RSV infection.

### PKR

Double-stranded RNA (dsRNA)-dependent protein kinase (PKR) is a serine/threonine kinase that exerts its own phosphorylation and the phosphorylation of other substrates, the most studied being the α subunit of the protein synthesis initiation factor eIF-2α. This process results in a shut-off of protein translation and inhibition of virus replication (Garcia et al., 2007). PKR is ubiquitous and constitutively expressed. PKR is induced in an inactive form by IFN and activated by binding to viral dsRNA. In addition to being phosphorylated, PKR was also identified as a target of ISGylation on Lys-69 and Lys-159 by ISG15 (Okumura et al., 2013) and SUMOylation on Lys-60, Lys-150, and Lys-440 (de la Cruz-Herrera et al., 2014). More recently, PKR Lys-256 was also shown to be modified by SUMO (El-Asmi et al., 2020a). SUMOylation as well as non-covalent SUMO interaction are required for PKR-dsRNA binding, PKR dimerization, eIF-2α phosphorylation and anti-VSV activity (de la Cruz-Herrera et al., 2014, 2017).

Remarkably, SUMO1 and SUMO3 expression exert a differential effect on PKR activation SUMO1 expression alone results in PKR and eIF-2α phosphorylation, whereas SUMO3 reduces PKR and eIF-2α phosphorylation upon viral infection or dsRNA transfection (Maarifi et al., 2018). Furthermore, the higher SUMO1-induced PKR activation is correlated with an inhibition of EMCV. Importantly, SUMO1 by inducing PKR activation in the absence of viral infection and SUMO3 by counteracting both PKR activation and stability upon EMCV infection shed a new light on the differential effects of SUMO paralogs (Maarifi et al., 2018). It is interesting to note that PKR ISGylation at Lys-69 and Lys-159, both located in the ds-RNA binding motif, by ISG15 also triggers PKR and eIF-2α phosphorylation in the absence of viral infection (Okumura et al., 2013). Cells expressing human PKR confer partial resistance to EMCV that is correlated with PKR and eIF-2α phosphorylation (Meurs et al., 1992). Therefore the stabilization of PKR by SUMO3 in response to IFN (El-Asmi et al., 2020a) could enhance its antiviral function.

### TRIM21

TRIM21 protein is an E3 ubiquitin ligase. The antiviral activity of TRIM21 varies among diverse viruses. Depletion of TRIM21 results in enhanced Coxsackievirus B3 (CVB3) replication, while its overexpression leads to increased IRF3 phosphorylation, increased IFNβ synthesis and reduced viral replication (Liu et al., 2018). More precisely, TRIM21 promotes IRF3 phosphorylation in infected cells by catalyzing the poly-ubiquitylation of MAVS, thereby enhancing type I IFN production. Interestingly, its implication in targeting viral proteins to regulate virus replication was recently shown (Mu et al., 2020). TRIM21 promotes the ubiquitylation and proteasomal degradation of HBV DNA polymerase using its RING finger domain, which has E3 ligase activity thus resulting in the restriction of HBV DNA replication (Mu et al., 2020).

### ISG15

The antiviral actions of ISG15 are mediated by unconjugated ISG15 and/or by ISGylated host and viral proteins (Morales and Lenschow, 2013; Perng and Lenschow, 2018; Freitas et al., 2020). Mice lacking ISG15 or the ISG15 E1 enzyme, ubiquitin-activating enzyme E1 homolog (UBE1L; also known as UBA7), are more susceptible to Sindbis virus, IAV and IBV (Lai et al., 2009; Morales et al., 2015; Perng and Lenschow, 2018). Also, it has been reported that the ISGylation of both host and viral proteins and the non-covalent binding of ISG15 to host proteins can disrupt viral replication (Perng and Lenschow, 2018).

Several studies showed that ISG15 functions as a critical antiviral molecule against many viruses including RSV, IAV, IBV, Sindbis virus, HSV-1, and HCV (Okumura et al., 2006; Lenschow et al., 2007; Hsiang et al., 2009; Kim and Yoo, 2010; Morales and Lenschow, 2013; Gonzalez-Sanz et al., 2016; Hermann and Bogunovic, 2017; Perng and Lenschow, 2018). Conversely, some viruses induce viral specific proteins that can deconjugate ISG15 from its target proteins or prevent the generation of ISGylated proteins, thus abrogating the antiviral response (Yuan and Krug, 2001; Frias-Staheli et al., 2007; Guerra et al., 2008). In contrast, deletion of Ubiquitin Specific Peptidase 18 (USP18), the major ISG15 specific protease, which counteracts ISG15 conjugation, increases ISGylation in cells and renders cells more resistant to viral infection in response to IFN (Perng and Lenschow, 2018), suggesting that ISGylation is essential for host defense against viral infections.

Interestingly, it has been reported that protein ISGylation enhances condensation of STAT1 and STAT2, and their association with PML NBs (Banani et al., 2016), suggesting that ISGylation contribute with PML NBs to the formation of a favorable nuclear environment for the expression of specific genes. Therefore, SUMOylation–ISGylation network could play a central role in the stabilization of ISG products and in enhanced IFN-induced antiviral defense.

In conclusion, increased poly-SUMOylation upregulates IFN-induced ISGylation and stabilizes in TRIM25-dependent manner the level of several restriction factors known to inhibit various steps of virus cycle. Despite these mechanistic insights, cellular signaling by poly-SUMO chains is still incompletely understood. Therefore, further studies are needed to validate and identify the SUMOylation sites of several stabilized ISG products such as GBP1, GBP5, Tetherin/BST2 and members of IFIM and IFIT families. Also, some of the stabilized restriction factors such as TRIM25, MxB, and SAMHD1 have been shown to be SUMOylated (El-Asmi et al., 2020a) though it is unknown whether their SUMOylation is required for their antiviral activities. Remarkably, by increasing poly-SUMOylation, IFNα targets both SUMO and ISG15 pathways. Indeed, IFNα enhances SUMOylation of Ubc9 and proteins implicated in ubiquitin pathway (Maroui et al., 2018), and also stabilizes ISG15 and proteins implicated in ISG15 pathway (El-Asmi et al., 2020a).

## Conclusion and Perspectives

ProMyelocytic leukemia protein, the organizer of PML NBs, is essential for IFN-enhanced global cellular SUMOylation and the SUMOylation of key players of IFN pathway. Its RNF4-dependent degradation later during IFN treatment suggests a negative regulation of SUMOylation and a return to cell homeostasis. Importantly, increased poly-SUMOylation in response to IFN upregulates both IFN-induced ISGylation and the expression of several restriction factors resulting in an enhanced IFN-induced antiviral state.

Multiple reports have demonstrated that SUMOylation and ISGylation are important for both intrinsic and innate immune antiviral responses. However, the biological significance of their cooperation in protein stability and antiviral defense is just emerging and necessitates further studies to clarify the role of the crosstalk between SUMOylation and ISGylation in intrinsic and innate immune responses. In this context, system-level identification of Ubl substrates using high sensitivity MS-based proteomic approaches is playing a key role to profile the changes in modification upon different cell stimuli and to identify acceptor sites.

The functions of poly-SUMO chains are growing. Recent findings showed that TRIM25 and RNF4, respectively, drive the SUMO3-dependent stabilization and destabilization of target proteins in response to IFNα. Whereas the ubiquitylation of poly-SUMO chains in proteasome-dependent protein degradation is well established, the ISGylation of poly-SUMO chains needs further experiments. UbcH8 and TRIM25 serve as E2- and E3-conjugating enzyme for both ubiquitin and ISG15, respectively. The identification of poly-SUMO-ISG15 chains and the determination whether TRIM25 and others Ubl conjugating enzyme could also act as a poly-SUMO-ISG15 ligase may play a key role in future discoveries of crosstalk between SUMOylation and ISGylation.

Further studies on the formation of hybrid chains ubiquitin-ISG15, SUMO-ubiquitin, SUMO-ISG15 and on the enzymes catalyzing their formation will confer an extra layer of complexity. The technological advances that led to the large-scale identification of Ubl substrates and their sites of modification provide important insights into Ubl functions. In particular, the identification of the acceptor sites, the sequence motifs, the types of branching, and the domains on which these modifications are located on target substrates in response to cell stimuli have significantly extend our understanding of Ubl regulation, The characterization of the interplay between SUMOylation, ubiquitylation and ISGylation pathways could help to identify antiviral targets, offering new opportunities to progress in antiviral defense mechanisms.

## Author Contributions

All authors listed have made a substantial, direct and intellectual contribution to the work, and approved it for publication.

## Conflict of Interest

The authors declare that the research was conducted in the absence of any commercial or financial relationships that could be construed as a potential conflict of interest.

## Abbreviations

CVB3: coxsackievirus B3
Daxx: death domain associated protein
DENV: dengue virus
GBP: guanylate binding protein
HIV-1: human immunodeficiency virus 1
HCV: hepatitis C virus
HSV-1: herpes simplex virus 1
IAV: influenza A virus
IBV: influenza B virus
IFN: interferon
IFI16: IFN γ inducible protein 16
IFITM: interferon-induced transmembrane proteins
IFIT: IFN-induced protein with tetratricopeptide repeats
ISGs: IFN-stimulated genes
KSHV: Kaposi’s sarcoma-associated herpesvirus
MLV: murine leukemia virus
Mx: myxovirus resistance protein
PKR: double-stranded RNA-dependent protein kinase
PML: ProMyelocytic leukemia protein
RSV: respiratory syncytial virus
SAMHD1: SAM domain and HD domain-containing
SAMD9: Sterile Alpha Motif containing domain 9
SUMO: Small Ubiquitin-related MOdifier
STAT: signal transducer and activator of transcription
TRIM: TRIpartite motif
VACV: vaccinia virus
LC-MS/MS: liquid chromatography-tandem mass spectrometry
Ni-NTA: nickel-nitrilotriacetic acid
Ubl: ubiquitin-like.
